# Poly(lactic acid) and Nanocrystalline Cellulose Methacrylated Particles for Preparation of Cryogelated and 3D-Printed Scaffolds for Tissue Engineering

**DOI:** 10.3390/polym15030651

**Published:** 2023-01-27

**Authors:** Mariia Leonovich, Viktor Korzhikov-Vlakh, Antonina Lavrentieva, Iliyana Pepelanova, Evgenia Korzhikova-Vlakh, Tatiana Tennikova

**Affiliations:** 1 Institute of Chemistry, Saint Petersburg State University, Peterhoff, Universitetskii pr. 26, 198504 Saint Petersburg, Russia; 2 Institute of Technical Chemistry, Gottfried-Wilhelm-Leibniz University of Hannover, 30167 Hannover, Germany; 3 Institute of Macromolecular Compounds, Russian Academy of Sciences, 199004 Saint Petersburg, Russia

**Keywords:** poly(lactic acid), nanocrystalline cellulose, methacrylation, particles, 3D printing, scaffolds, tissue engineering

## Abstract

Different parts of bones possess different properties, such as the capacity for remodeling cell content, porosity, and protein composition. For various traumatic or surgical tissue defects, the application of tissue-engineered constructs seems to be a promising strategy. Despite significant research efforts, such constructs are still rarely available in the clinic. One of the reasons is the lack of resorbable materials, whose properties can be adjusted according to the intended tissue or tissue contacts. Here, we present our first results on the development of a toolbox, by which the scaffolds with easily tunable mechanical and biological properties could be prepared. Biodegradable poly(lactic acid) and nanocrystalline cellulose methacrylated particles were obtained, characterized, and used for preparation of three-dimensional scaffolds via cryogelation and 3D printing approaches. The composition of particles-based ink for 3D printing was optimized in order to allow formation of stable materials. Both the modified-particle cytotoxicity and the matrix-supported cell adhesion were evaluated and visualized in order to confirm the perspectives of materials application.

## 1. Introduction

Bone loss and fracture caused by disease or injury re considered major health problems [1]. Today, the most common method to address bone defects is bone grafting, but unfortunately, it is still problematic for clinicians to choose between autografts, allografts or engineered tissues [2]. Recently, bone tissue engineering (BTE) has gained more attention as a potential treatment for bone defects. The strategy for bone tissue engineering involves the following steps: (1) identification of a suitable cell source, isolation of cells and expansion of cells to sufficient amounts; (2) obtaining of a biocompatible material that can be used as a cell substrate and processed into the required shape (scaffold); (3) seeding of the scaffold with cells, which can then be cultivated in bioreactors; (4) placement of the material-cell construct into the target site in vivo [2]. In this regard, scaffolds are the key component of BTE, as they regulate bone healing and mimic the extracellular matrix (ECM) function in the bone tissue [2,3]. An ideal scaffold for bone tissue repair should have a three-dimensional porous structure with a highly interconnected pore network, being biocompatible and controllably bioresorbable [3,4]. The ideal strategy for regenerative bone therapies is to use a tunable scaffolding material that can modulate the process of healing while providing mechanical support [2,5]. 

Tuning of scaffolding material properties requires a certain level of versatility, which could be provided by polymers—the intelligent choice of macromolecules—and their combinations allow the formation of materials with a wide variety of both mechanical and biological properties [6]. The development of composites allows further upgrades the level of the materials’ versatility [7]. However, despite the great progress in the preparation of various materials for scaffold formation, there are still unsolved problems, such as control over spatial and temporal distribution of bioactive molecules, as well as the formation of gradients [8,9,10]. It is now obvious that successful scaffold application requires control over its micro/nanotopology as well as providing the scaffold with the ability to promote and guide cell-induced tissue regeneration through the regulation of local microenvironment by exposing the appropriate signals at the desired site for the required time frame [11]. The development of such complex scaffolds is essential, because the combination/distribution of various materials could resemble the complexity of natural bone tissue. These issues require higher levels of material versatility, which could be provided by the application of combinations of polymeric particles of different origins with different properties as “ink” for the 3D printing of scaffolds. Particles possessing different chemical natures and various densities and surface properties can be aggregated into supermacroporous matrices using different combinations of such particles, which should allow the tuning of the mechanical properties and surface topology of the pores [12,13]. It could be supposed, for example, that increasing the number of hard particles and the density of cross-linking should result in greater mechanical strength, while the addition of soft particles should favor the formation of gel-like materials. Particles with different properties could be combined into one material to mimic the tissue contacts; for example, osteochondral scaffolds [14]. Moreover, the useful peculiarities of the particles are their potential for surface modification and their ability to entrap different substances into their inner volume, which allow the spatial distribution of osteogenic or angiogenic factors and their temporally controlled release [15]. The release of various drugs could also be very useful in the cases when bone regeneration is associated with surgical interventions due to various diseases [16].

There are many methods that were developed in order to obtain porous materials applicable for supporting 3D cell growth: melt-molding, fiber bonding, porogen leaching, gas foaming and phase separation [17]. Pore diameter greater than 50 μm, pore uniformity and interconnectivity are the main goals for these methods. However, not all these approaches are suitable for preparation of particles-based scaffolds. One of the interesting techniques that is useful for the preparation of supermacroporous matrices is cryogelation. This method is based on solid–liquid phase separation, and includes freezing of the solution or preformed gel, cross-linking interactions between macromolecules or particles and removal of the solvent [18]. As the solution is frozen, the solvent crystals grow until they come into contact with other crystals. Thus, the solvent crystals form a common extended structure. The phase containing the polymer or particles is concentrated around the crystals and is called the unfrozen liquid microphase (ULMP). In fact, crystals of water push the polymer molecules or particles together. This process is called ”cryo-concentration“ [18]. In such concentrated conditions, different chemical cross-linking reactions, such as cryo-polymerization, could run very efficiently, even at temperatures below 0 °C [19]. The last stage of cryogel formation is the removal of the solvent crystals via freeze-drying, leaving the supermacroporous structure, which is useful for cell seeding and proliferation (see the scheme and images in [19]). 

Previously developed 3D printing techniques are also very good candidates for the preparation of scaffolds by particle controllable aggregation. Modern 3D printers allow precise control over the material composition as well as simultaneous combination of different printing techniques: [20,21,22,23]. However, 3D organization of particles into scaffolds via direct ink writing (DIW) and stereolithography (SLA) is still an unexplored area [24]. Wide application of such 3D printing techniques in clinics with utilization of particles as versatile material for scaffold formation could help to solve the problems of auto- and allograft applications, which comprise a shortage of bone, donor site morbidity and additional operations on the patients [2].

Poly(lactic acid) (PLA) is one of the most widely used polymers for biomedical applications [25]. This polyester is often a good selection in the case of scaffolds for BTE [23,26]. PLA is biocompatible and its application in medicine is approved by the Food and Drug Administration (FDA) agency [25,27]. One of the most valuable features of PLA is its controllable biodegradability [27]. PLA is a thermoplastic polymer that is non-soluble in water and relatively hydrophobic [25]. On one hand, this allows the formation of stable biomaterials with different geometries, but on the other hand, this could cause unpredictable in vivo biointeractions between PLA-based materials and the surrounding tissues [28]. In order to improve the mechanical properties and hydrophilicity of PLA-based scaffolds, the PLA is commonly combined with various particles, such as hydroxyapatite [29], chitosan [30,31], graphene oxide [32] etc., in order to form composite scaffolds. Recently it was recognized that among the prospective additives for PLA are micro- or nanocrystalline cellulose (MCC/NCC) particles, which are very biocompatible and can significantly increase scaffold mechanical performance and hydrophilicity [33]. NCC particle surfaces could be also chemically modified with special molecules in order to affect the biological properties of the polyester-based composite scaffold; for example, inducing calcification and bone tissue formation around the implanted material [34,35]. Such composite scaffolds could be considered as “particle-incorporating scaffolds”, in which particles are dispersed into a PLA-based continuous phase. This type of composite scaffold is often prepared via 3D printing, which uses fused deposition modelling (FDM) technology [24,34]. Despite significant research in this area, the approach presents challenges with regard to control over biomolecule delivery, cell infiltration and viability within the scaffold matrix and clinical handling [36]. Moreover, it is difficult to use FDM technology only for spatial control of component distribution. 

**Figure 1:**
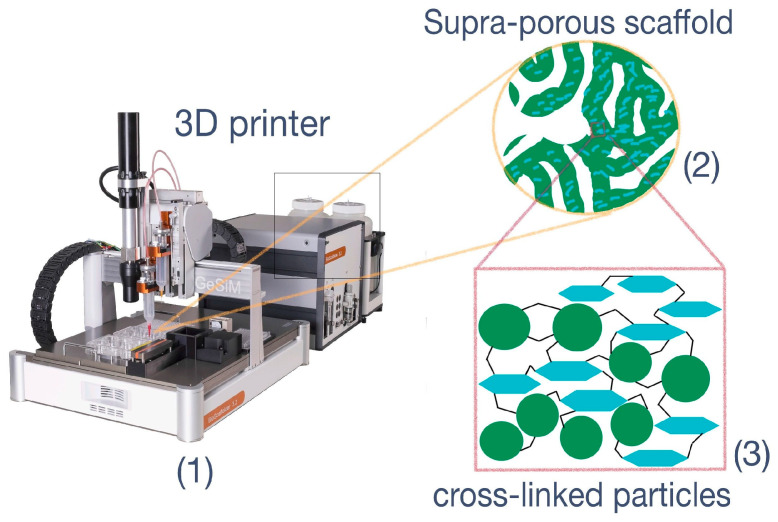
The idea of particle-based scaffold formation with application of DIW + SLA 3D-printing technologies. (**1**) 3D-printer supplied with extruder-type printing head and UV-lamp; (**2**) supermacroporous matrix with interconnected pores, which could serve as scaffold; (**3**) covalently cross-linked particles. The green circles represent PLA particles, and the turquoise hexagons indicate NCC particles.

Another possible type of particle organization in materials for tissue engineering is the “particle-based scaffold”. In such a structure, PLA microspheres themselves or their combinations with other particles represent the building blocks for scaffold framework formation [37,38]. It is well-known that crude PLA can be easily converted into microspheres via emulsion protocols [39]. In previous studies, microspheres based on PLA or other polyesters were used as blocks for preparation of scaffolds via interfusion of particles, which was provoked by thermal sintering [14,40] or solvent-induced sintering [41] protocol. Additionally, the selective laser sintering (SLS) technique could be successfully applied to produce nanocomposite PLA-hydroxyapatite scaffolds [42]. The mentioned techniques of particle-based scaffolds preparation are very similar to the procedures of powder-based technologies. In fact, none of these techniques allow reliable preservation of encapsulated biomolecule stability, due to the application of elevated temperatures, laser, or organic solvents. In this regard, the simultaneous application of soft DIW and SLA 3D printing techniques is of interest because it allows the formation of a scaffold with spatial distribution of different particle types within the scaffold (Figure 1). These techniques also allow the formation of so-called gradient scaffolds.

To the best of our knowledge, no studies on PLA and NCC particle controllable organization into scaffolds via covalent cross-linking has been previously published. Thus, the purpose of this study was investigation of the unexplored possibility of PLA and NCC nanoparticle (NPs) covalent interaction to allow the production of 3D matrices, which could be used as scaffolds for tissue engineering. To provide the possibility of their chemical cross-linking, both PLA and NCC NPs were methacrylated (PLA-methacrylate—PLA-MA and NCC-methacrylate—NCC-MA). The effect of particle modification on the viability of cells was tested. Then, we aimed to investigate the possibility of chemical interparticle cross-linking without external cross-linkers, as well as in the presence of bifunctional (poly(ethylene glycol) dimethacrylate) and multifunctional (gelatin methacrylate) macromolecular cross-linkers. The ability of methacrylated particles to form 3D structures was firstly analyzed via rheological measurements, and then optimized mixtures were used for porous matrix formation. Cryogelation and 3D-printing approaches were applied for this purpose, allowing comparison of materials obtained by these methods. The effect of composition used for matrix preparation on cell attachment was finally tested.

## 2. Materials and Methods

### 2.1. Materials

All monomers, initiators, and agents for modification, as well as nonionic poly(oxyethylene-*b*-oxypropylene) (Pluronic F-68, M_W_ = 138.16), sodium dodecyl sulfate (SDS), poly(vinyl alcohol) (PVA, M_W_ = 70,000), 2-hydroxy-4′-(2-hydroxyethoxy)-2-methylpropiophenone (Irgacure 2959) and poly(ethylene glycol) dimethacrylate (PEGDA, M_W_ = 700) were purchased from Sigma-Aldrich (Munich, Germany). NCC was a product of Blue Goose Biorefineries Inc. (Saskatoon, SK, Canada). Organic solvents (toluene, chloroform, methanol, etc.) used for polymer synthesis and modification were from Vecton (St. Petersburg, Russia). All organic solvents were distilled before application. Poly(L-lactic acid) (PLA, M_W_ = 18,500; Ð = 1.23) was synthesized by ring-opening polymerization of L-lactide (Sigma-Aldrich, Munich, Germany) as previously described [43]. Methacrylated gelatin (GelMA) was synthesized as described in [44].

Dulbecco’s Modified Eagle Medium (DMEM-F12) (Biolot, Saint Petersburg, Russia)/10% fetal bovine serum (FBS) (Biowest, Riverside, MO, USA)/50 IU/mL penicillin/50 µg/mL streptomycin (Biolot, Saint Petersburg, Russia) was used for cell culture experiments.

### 2.2. Cells

MSCs (bone marrow mesenchymal stem cells) and NIH 3T3 (mouse fibroblast cells) cell lines were obtained from the German Collection of Microorganisms and Cell Culture (DSMZ, Braunschweig, Germany). 

### 2.3. Methods

#### 2.3.1. PLA-Based Particle Formation

PLA-based particles were obtained by single emulsion method as previously described [39,45]. Shortly, 2 mL solution of PLA in dichloromethane (50 mg/mL) was dispersed into 20 mL of ice-cold water phase, in which 0.2 g of SDS (1 wt%) and 0.6 g of Pluronic F-68 (3 wt%) were preliminarily dissolved. The emulsion was formed via simultaneous application of ultrasound homogenizer (Sonopuls HD2070, Bandelin, Berlin, Germany) and magnetic stirrer (MR Hei-Mix S, Heidolph, Schwabach, Germany) at 700 rpm during 10 min. The resulting emulsion was diluted in 80 mL of ice-cold 1 wt% PVA aqueous solution. Dichloromethane was removed by evaporation using rotary evaporator (Hei-VAP Precision ML/G3B, Heidolph, Schwabach, Germany) at 100 mbar for 2–3 h. The obtained particles were isolated by centrifugation (Sigma 2–16 KL, Sigma, Darmstadt, Germany) at 10,000 g and washed three times with distilled water. 

#### 2.3.2. PLA Particle Modification with 2-Aminoethyl Methacrylate

First, the available reagent 2-aminoethyl methacrylate in hydrochloride form (2-AEMA●HCI) was converted to a free base by treatment with diisopropylethylamine. For that 0.3 g of 2-AEMA●HCI was placed in a 25 mL vial, 15 mL of diethyl ether was added and 450 μL of diisopropylethylamine was added at stirring. The reaction proceeded for 2 h under vigorous stirring at room temperature. Then the precipitate was separated by centrifugation, and the supernatant containing 2-AEMA was concentrated on a rotary evaporator. As a result, the free base 2-AEMA as an oil was isolated from the crystalline hydrochloride form of 2-AEMA●HCI.

**Figure 2:**
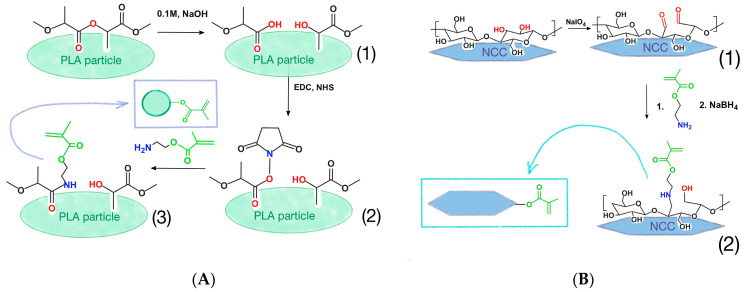
The schemes of methacrylated particles formation via reaction with 2-aminoethylmethacrylate: (**A**)—modification of PLA particles; (**B**)—modification of NCC particles.

The PLA particle modification scheme is shown in Figure 2A. A quantity of 500 mg of PLA particles were treated with 0.1 M NaOH solution (50 mL) to generate carboxyl groups (Figure 2(A1)) on the particle surface [46]. The hydrolysis was conducted for 30 min at room temperature, under stirring (350 rpm). Then, the particles were purified 5 times by Vivaspin-column dialysis (filter MWCO 10,000; 25 mL; Merck, Darmstadt, Germany) against 20 mL of ice-cold grade water (MQ water) and 3 times against 0.05 M buffer solution of 2-(N-morpholino)ethanesulfonic acid, pH 5.6 (MES buffer). 

The suspension of particles in 0.05 M MES with a pH of 5.6 was precooled down to 5 °C, then the solution of 30 mg N-hydroxysuccinimide (NHS) and 20 mg of (N-3-dimethylaminopropyl)-N-ethylcarbodiimide hydrochloride (EDC) in the same buffer were added consecutively to activate the carboxyl groups (Figure 2(A2)) on the particle surface. After that, the particles were purified 5 times by Vivaspin-column dialysis (MWCO 10,000) against MQ water and 3 times against 0.1 M borate buffer solution (BBS, pH 8.4).

Next, 200 mg of AEMA in a 0.1 M borate buffer solution (pH 8.4) was added to the particle suspension. The reaction was performed for 3 h at room temperature while stirring at 500 rpm to obtain the final product—methacrylated PLA particles (PLA-MA, Figure 2(A3)). Then, the particles were purified by Vivaspin-column dialysis (MWCO 1000) against ultrapure water.

#### 2.3.3. Nanocrystalline Cellulose Modification with AEMA

The PLA particle modification scheme is shown on Figure 2B. A quantity of 1 g of 8% stock nanocrystalline cellulose (NCC) suspension was diluted 10 times with MQ water and cooled to 5 °C while stirring for 30 min. Then, 500 mg of sodium metaperiodate in 5 mL of water was added into the reaction mixture. The periodate-containing mixture was wrapped in aluminum foil to avoid light exposure. The reaction mixture was gently stirred at 5 °C in the dark for 24 h. Thereafter, the obtained 2,3-dialdehyde NCC (DA-NCC, Figure 2(B1)) was purified by Vivaspin-column dialysis (MWCO 1000) against MQ water. 

Then, 300 mg of AEMA in 6 mL of MQ water was added to 100 mL of DA-NCC (1.0 g) suspension in 0.01 M BBS (pH 8.4). The reaction was carried out for 24 h at 20 °C under vigorous stirring at 500 rpm. After that, 300 mg of sodium borohydride was added to the reaction mixture to reduce the remaining aldehyde groups and imine bonds. The reaction was carried out for 24 h at 20 °C under stirring at 500 rpm. The product (Figure 2(B2)) was purified by Vivaspin-column dialysis (MWCO 1000) against MQ water.

#### 2.3.4. Particle Characterization

The introduction of methacrylate (MA) groups onto the particle surfaces was proved by: (1) ^1^H NMR spectra, which were recorded with equipment of Magnetic Resonance Research Centre of St. Petersburg State University (Bruker Avance spectrometer, 400.13 MHz) in CDCl_3_; (2) FTIR spectra, which were recorded from 4000–400 cm^−1^ in KBr tablets with application of equipment of Chemical Analysis and Materials Research Centre of St. Petersburg State University (IRAffinity-1, Shimadzu Corporation, Kyoto, Japan).

Particle hydrodynamic diameter and ζ-potential were measured with Zetasizer Nano ZS (Malvern, Enigma Business Park, UK) equipped with a He–Ne laser beam at λ = 633 nm. Nanoparticle tracking analysis (NTA) was performed with a NanoSight NS300 particle analyzer (Malvern, Enigma Business Park, UK).

The morphology of particles was detected by TEM with equipment from the Centre for Molecular and Cell Technologies of St. Petersburg State University—Jeol JEM-1400 STEM (Tokyo, Japan). SEM was used for the investigation of particles and matrix morphology and this equipment was provided by Interdisciplinary Resource Centre for Nanotechnology of St. Petersburg State University—Zeiss Supra 40VP (Carl Zeiss MicroImaging GmbH, Oberkochen, Germany).

#### 2.3.5. Rheological Measurements of Particle Cross-Linking

The UV photo-curing of methacrylated particle suspensions was investigated at 25 °C using a MCR 302 Modular Rheometer (Anton Paar, Graz, Austria) equipped with a plate-plate geometry (20 mm diameter, 0.3 mm gap size) and by performing in situ UV cross-linking by irradiation with a UV lamp (365 nm, 75 W/cm^2^, Delolux 80, Delo, Windach, Germany) from below. The crosslinking kinetics was recorded with a time sweep oscillatory test under constant strain amplitude of 1% and at a constant frequency of 1 Hz, which is within the linear viscoelastic (LVE) region.

A series of samples of nanoparticle suspensions of different compositions (PLA-MA/NCC-MA: 80:20, 60:40, 40:60, 20:80 wt/wt; PLA-MA/NCC-MA 80:20 wt/wt + 20 wt% PEGDA or GelMA) were prepared. A quantity of 1 mg of photoinitiator (0.1 wt/vol %, Irgacure 2959) was added to the 1 mL of prepared mixture. The concentration of particles in the suspension was varied (10, 50 and 100 mg/mL). A 400 μL sample was placed between the two rheometer plates using an automatic pipette. The sample was irradiated with UV radiation in situ; the irradiation time was 10 min. Then, the rheological measurements were continued for a further 10 min without irradiation.

#### 2.3.6. Cryogelation

Matrices were formed in a 1 mL syringe. A quantity of 0.8 mL of particle aqueous suspension containing 64 mg of PLA-MA and 16 mg of NCC-MA was prepared, and 0.15 mL of solution containing 216 mg of cross-linking agent (20 wt% from the total mass of reaction mixture, PEGDA or GelMA) was added to the particle suspension and homogenized with a thermoshaker (TS-100, Biosan, Riga, Latvia) at 500 rpm during 5 min. Then, solutions of 1.3 mg of initiator—ammonium peroxodisulfate (APS)—and 1.5 mg of reaction activator N,N,N′,N′-tetramethylethylenediamine (TMEDA) in 250 µL of ultrapure water were prepared. First APS and then TMEDA solutions were added under vigorous stirring to the precooled to 5 °C suspensions of particles with cross-linker. After that, the reaction mixture was immediately transferred into the syringe and placed in the freezer at −13 °C for 24 h. After this period, the samples were allowed to thaw, and the matrices were washed by passing a 100-fold of the volume of MQ water through them. Then, syringes were gently cut open to release the resulting matrix, which was further washed with MQ water over 48 h at 150 rpm stirring with thermoshaker.

#### 2.3.7. 3D Printing

3D CAD models of the desired object for printing were designed and the necessary settings were lined up using Autodesk Inventor Professional 2015 software (Autodesk Inc., San Rafael, CA, USA). 3D printer Allevi 1 (Allevi Inc., Philadelphia, PA, USA) and GeSim Bioscaffolder 3D printer (Radeberg, Germany) were used for 3D printing. These printers were equipped with extrusion-type print head and LED 365 nm (1.5 W/cm^2^) UV lamp for photo-cross-linking of the material.

Matrices were printed in the wells of 24-well plates using a mixture of particle suspension with added cross-linking in a composition similar to that described above (see Section 2.3.6). Printing parameters were optimized: printing needle diameter, pressure, and printing speed. In order to avoid gel formation, the optimal printing suspension temperature of 37 °C was also selected when working with the sample containing GelMA as an additional cross-linking agent. Uniform extrusion was observed at the printing needle diameter 0.254 and length 6.35 mm, pressure (0.5 PSI or 3.45 kPa). The optimum printing speed was 5 mm/s. 

Before printing 10 µL (11 µg/µL) of photoinitiator (Irgacure 2959) solution in ethanol was added to the mixture. The suspension was loaded into an extruder cartridge with pre-screwed print heads of the required diameter. The cross-linking of the sample was performed in layers: after the printing of the first level of the matrix, the UV lamp was turned on, the sample was irradiated for 10 min, and then the printing process was resumed. Finally, printed samples (length/width/height = 1/1/0.4 cm) were left for an hour in order to allow the radical cross-linking reaction to proceed, as well as for drying of the samples.

#### 2.3.8. Study of Mechanical Properties

The samples of cryogels and 3D printed materials were prepared in the form of unified discs 5 mm in diameter and 5 mm thick. The mechanical analysis of the samples was performed by uniaxial compression tests on a Shimadzu EZTest EZ-L (Shimadzu Corporation, Kyoto, Japan) tensile testing machine. To ensure tight surface contact with the sample, the initial force was 0.1 N; the rate of motion was 2 mm/min. The series of parallel measurements (*n* = 3) were performed for all cryogel specimens. 

#### 2.3.9. Cell Culture Experiments

Cytotoxicity of particles. The cytotoxicity of the particles was studied using the CellTiter-Blue test (CTB), which is based on the ability of living cells to reduce the blue nonfluorescent reagent CTB-resazurin (7-hydroxy-3H-phenoxazine-3-one-10-oxide) to the pink fluorescent resorufin. 

A total of 8 × 10^3^ cells (MSCs) in 100 μL of culture medium were seeded in each well of a 96-well plate and cultured for 24 h (37 °C, 5% CO_2_, 80% humidity). Then, the culture medium was removed using a sterile glass Pasteur pipette and 200 μL of culture medium containing nanoparticles in the concentration range of 1–10 mg/mL was added. 

The cells were incubated in a CO_2_ incubator for 24/48/72 h at 37 °C, then the medium was removed and 100 μL of CTB solution in basal medium (1:10 (vol/vol)) was added to each well. The cells were incubated for another 2 h at 37 °C. The formation of a fluorescent product proportional to the number of viable cells was monitored fluorometrically (λ_ex_ = 544 nm and λ_emm_ = 590 nm). The data were normalized as a percentage of the control (wells containing cells incubated without test substances). The analysis was repeated five times for each concentration.

Cells adhesion. Before the cell culture experiments, the samples obtained by 3D-printing and cryogelation were washed with ultrapure water while stirring to remove unreacted components. Then they were sterilized with UV light (wavelength range: 200–295 nm) for 30 min on each side. 

MSCs were used in the experiments. A total of 4 × 10^4^ cells were seeded on the surface of matrices in 500 μL of nutrient medium in each well. The plate was then placed in a CO_2_ incubator (37 °C, 5% CO_2_, 80% humidity) for a predetermined period of time. Then cells attached to the cryogel surface were visualized. Staining was performed using DAPI fluorescent dye. For this purpose, the matrices were washed twice with warm 0.01 M phosphate-salt buffer solution (pH 7.4) for 30 min. Then, 200 μL of cold 4% paraformaldehyde in phosphate-salt buffer solution was added to the sample to fix the cells, and was left for 20 min at room temperature. After fixation, the samples were washed again with 0.01 M phosphate-salt buffer solution (pH 7.4), and the cell nuclei were stained for 15 min with DAPI working solution (1:1000 (vol/vol) in phosphate-salt buffer solution) at room temperature (22 °C) in the dark. The samples were analyzed using a fluorescence microscope (Olympus, BX-51 Olympus Corporation, Tokyo, Japan). The data from these experiments are presented as average ± SD (*n* = 5).

## 3. Results and Discussion

### 3.1. Preparation of Particles Capable of Participation in Free-Radical Polymerization-Mediated Cross-Linking

The idea of this study was to use PLA and NCC particles for the production of particle-based 3D scaffolds. PLA NPs were obtained via single emulsion protocol, while NCC particles were used as purchased. In order to provide the particles with the ability to participate in the free-radical cross-linking process, the methacrylate groups were introduced onto particle surfaces to obtain PLA-methacrylate (PLA-MA) and NCC-methacrylate (NCC-MA). 

In the case of PLA, we applied the previously developed protocol of PLA particle surface partial hydrolysis [46], which results in the formation of carboxylic groups (Figure 2A). These groups were then transformed into the activated esters and put into reaction with 2-aminoethylmethacrylate (AEMA) to obtain PLA-MA particles. In order to introduce methacrylate groups on the surfaces of the NCC particles, they were partially oxidized with sodium periodate. The formed aldehyde groups easily reacted with amino groups of AEMA to give NCC-MA (Figure 2B).

**Figure 3:**
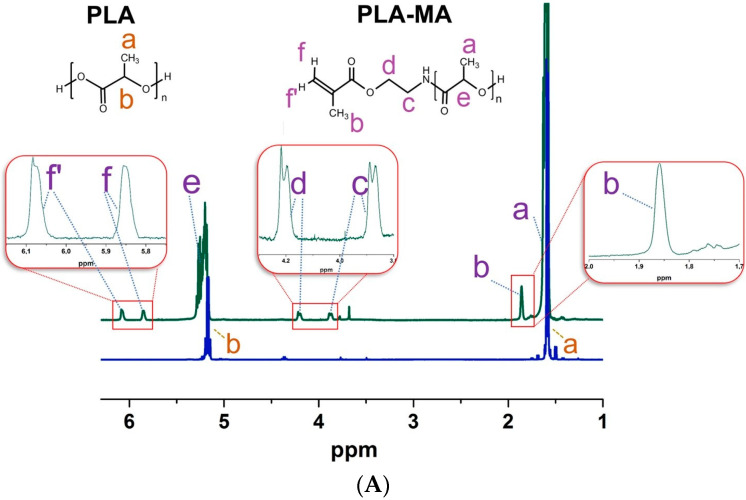

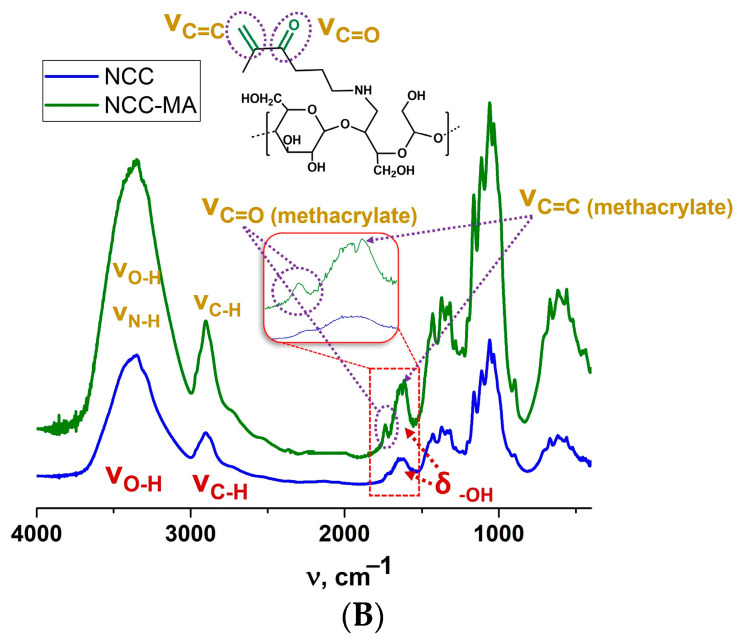
The evidence for successful introduction of methacrylate groups into particles under study: (**A**) ^1^H NMR spectra of PLA and PLA-MA particles dissolved in CDCl_3_; (**B**) FTIR spectra of NCC and NCC-MA.

The successful introduction of methacrylate moieties was proved by ^1^H NMR and FTIR spectra for PLA-MA and NCC-MA, respectively (Figure 3). In the ^1^H NMR spectrum of PLA-MA particles dissolved in CDCl_3,_ the signals corresponding to the presence of diastereotopic protons of methacrylate double bond (Figure 3, signals *f* and *f’*), protons of methylene groups between ester and amino groups (Figure 3, signals *d* and *c*) and protons of methacrylate methyl group (Figure 3, signal *b*) were found. 

Because of the non-solubility of NCC, the presence of methacrylate groups in modified NCC was analyzed by FTIR. In the spectrum of raw NCC, the absorption bands at 3357 cm^−1^ and around 2908 cm^−1^ were attributed to the O-H and C-H stretching vibrations, respectively. The absorption band at 1642 cm^−1^ could be attributed to the O-H vibration of absorbed water [47,48,49]. The bands corresponding to the C-H and C-O vibrations, contained in the polysaccharide rings of cellulose, were around 1374 cm^−1^. The vibration of C-O-C in pyranose ring was indicated by the absorption band at 1060 cm^−1^. In the NCC-MA spectrum, it was possible to observe the appearance of new bands. The band at 1738 cm^−1^ corresponded to the C=O stretching vibrations. It was also possible to observe an enhancement of intensity and sharpness of the band at 1617 cm^−1^, which could be due to the C=CH_2_ out-of-plane bending vibrations.

The Raman spectra of PLA and PLA-MA were recorded in order to check the presence of methacrylate bonds in modified PLA particles (see Supplementary Materials, Figure S1). In the presented spectrum of the modified particles, there were valent vibrations of the C=C bond at 1650 and 1626 cm^−1^, while in the spectrum of non-modified PLA, no signals were observed in this region.

Initial and modified particles were characterized by dynamic and electrophoretic light scattering (DLS and ELS), nanoparticle tracking analysis (NTA), and electron microscopy (TEM/SEM) (Table 1). The obtained data are presented in Table 1, and the data obtained by different methods are in good agreement with each other. One can observe that the initial PLA particles possessed greater diameter than that of raw NCC. Both PLA and NCC had negative ζ-potential. The value of this parameter was large enough to stabilize particle suspensions. Modification of particles by MA groups led to some growth in particle size and a slight decrease in surface charge. However, these changes were not drastic. It is obvious that growth of particle size could not be caused by particle enlargement due to the attachment of MA groups, but most probably resulted from particle aggregation due to the introduction of hydrophobic MA moieties. The increased width of particle size distribution after modification favors this statement (see Supplementary Materials, Figure S2). It should be noted that D_H_ of NCC particles is averaged as if they were spherical particles, although they were not.

**Figure 4:**
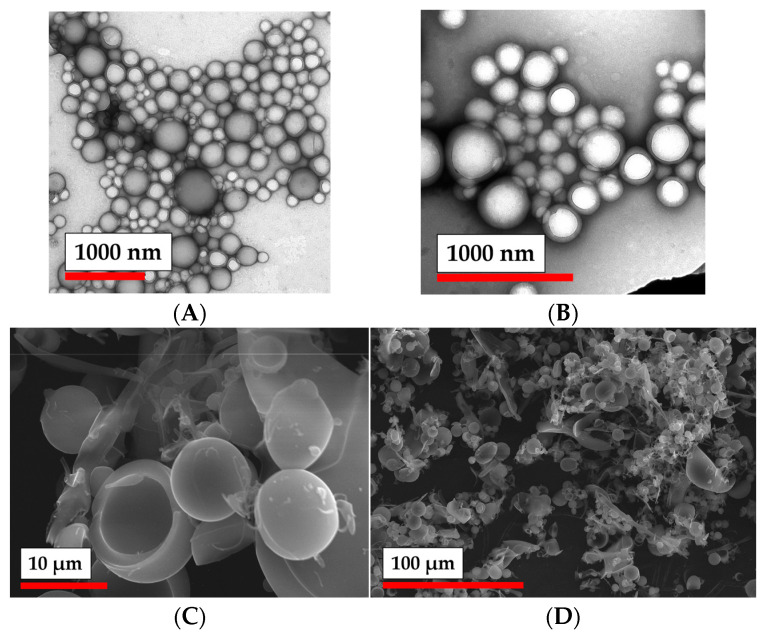
Morphology of PLA particles: (**A**) TEM microphotographs of initial PLA NPs; (**B**) TEM microphotographs of PLA NPs modified with AEMA; (**C**,**D**) SEM microphotographs of PLA NPs modified with AEMA.

The morphology of the modified particles was analyzed by TEM and SEM. One can observe that both PLA and PLA-MA (Figure 4) possessed round-shaped particles. Modification of PLA particles with MA groups did not seem to change their morphological features. However, these particles seemed to be quite prone to aggregation, which is also supported by the data obtained by nanoparticle tracking analysis (Figure S2, Supplementary Materials).

**Figure 5:**
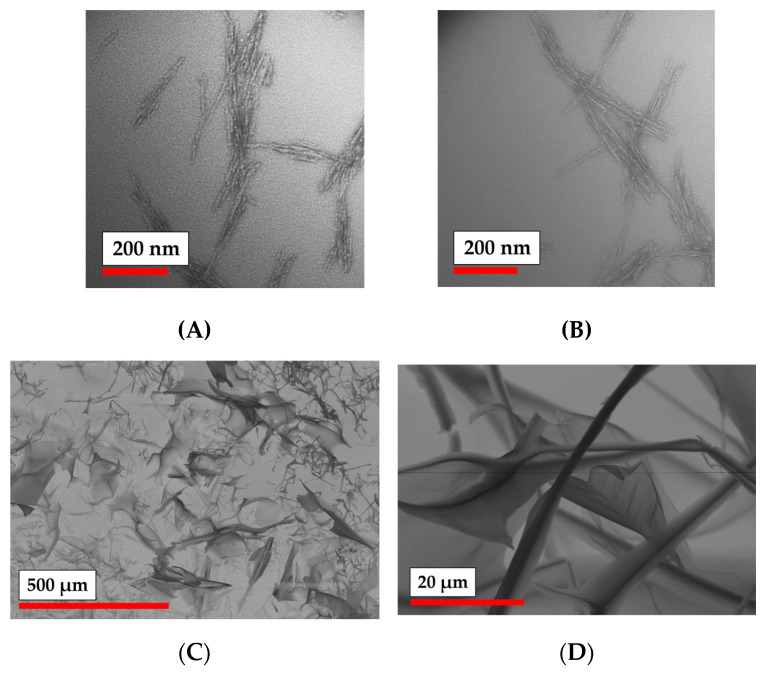
Morphology of NCC particles: (**A**) TEM images of non-modified NCC particles; (**B**) TEM images of NCC-MA particles; (**C**) SEM microphotographs NCC modified with AEMA; (**D**) SEM microphotographs NCC modified with AEMA.

NCC-MA particles appear on the TEM images in the form of needles (Figure 5). The obtained sample of NCC-MA was observed by SEM, as presented in Figure 5. One can see the anisotropic character of the sample, which seems to reflect the organization of the NCC-MA particles into the fibrillary structures. The organization of NCC into such structures is well-known as also being the case for non-modified NCC [50].

The study of nanoparticle morphology can be summarized by the observation that the modification of nanoparticles does not affect their morphological features, but possibly could lead to their more intensive aggregation. Additionally, the size of the particles grew a little after the attachment of MA groups.

### 3.2. Effect of Particle Modification on Cells Viability

**Figure 6:**
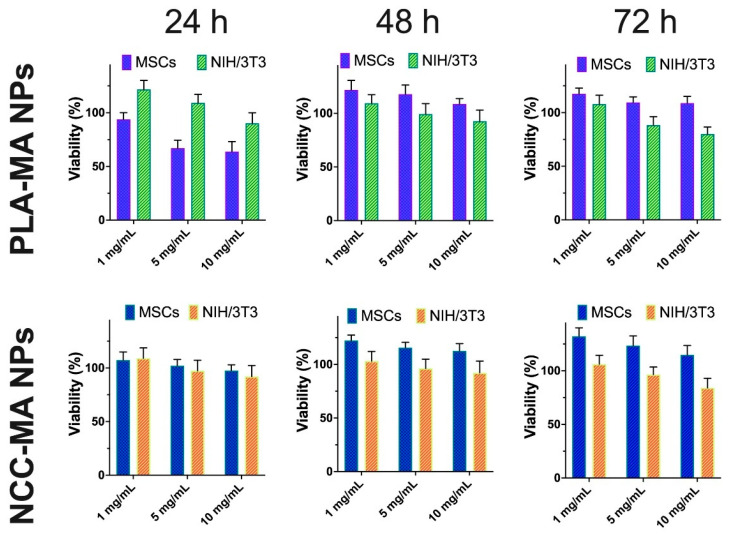
The effect of PLA-MA and NCC-MA particles on cell viability. MSCs and NIH/3T3 were exposed to the indicated concentrations of particles for 24, 48 and 72 h. Viability (%) is referred to the control, which was pure media without particles. Data are presented as mean ± SD (*n* = 5).

It is well known that PLA and NCC particles are quite biocompatible materials [35,46]. Here we were aimed to evaluate the effect of methacrylic groups attachment to the surface of such particles on their cytotoxicity. Two types of cells, which were used in this study, namely bone marrow mesenchymal stem cells (MSCs) and mouse embryonic fibroblasts (NIH/3T3), were widely used in tissue engineering related research. Different concentrations of particles were added to the cultured cells, and cell viability was evaluated via MTT assay after different periods of co-incubation (Figure 6). 

The obtained results clearly show that both methacrylated types of particles (PLA-MA and NCC-MA) did not greatly affect cells viability at concentrations 1 and 5 mg/mL. There was a certain decrease in viability in the case of NIH/3T3 cells after 72 h of 10 mg/mL for both types of particles co-incubated with cells. However, no such effect was observed for MSCs. 

In general, it might be concluded that modification of PLA and NCC NPs by methacrylate groups does not result in a drastic increase in their toxicity. The obtained particles could be considered as quite non-toxic and recommended for further studies on their possible application for scaffolds formation.

### 3.3. Rheological Studies of PLA and NCC Particle Cross-Linking

The study of the possibility of particle interaction under free-radical process conditions was the key stage of this research. For that, the rheology experiments were applied to detect the appropriate particle suspension concentration and PLA-MA/NCC-MA ratio at which the storage modulus (G’) would increase, indicating the formation of a three-dimensional network. Particle suspensions of different compositions together with photo-initiators were placed between the two rheometer plates (Supplementary Materials, Figure S3). The rheological measurements were started simultaneously with sample UV irradiation to detect the growth of G’. The exposure to UV continued over 10 min and the measurement process after that period was conducted without irradiation. Thus, we also detected the effect of post-exposure cross-linking on the rheological properties of the system.

**Figure 7:**
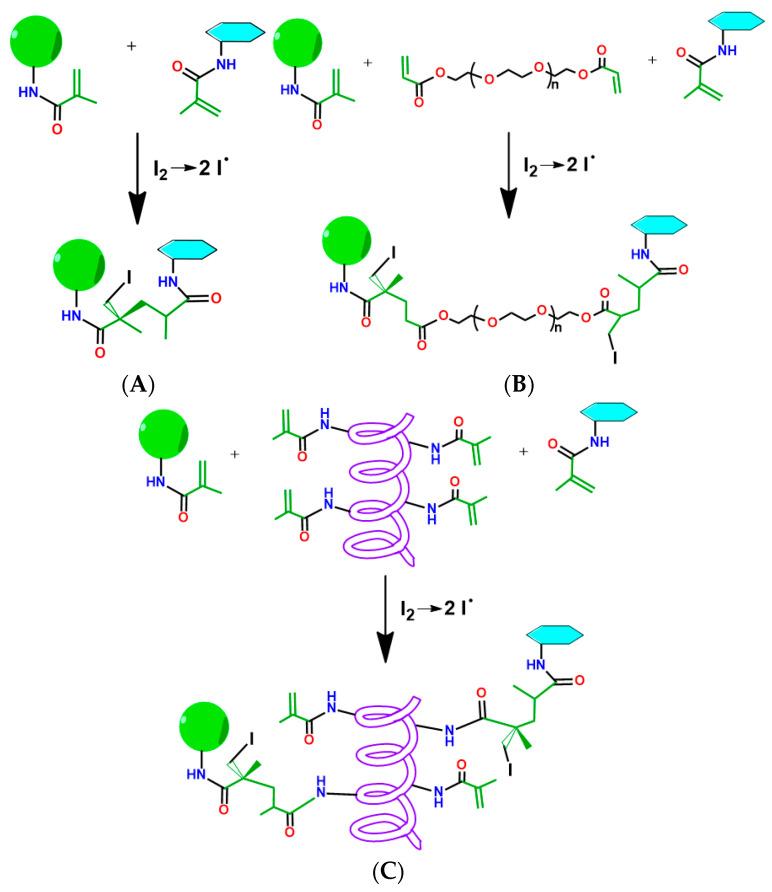
The possible schemes of particle cross-linking: (**A**) direct methacrylated particle cross-linking; (**B**) particle cross-linking with application of PEGDA as cross-linking agent; (**C**) particle cross-linking with application of GelMA as cross-linking agent.

**Figure 8:**
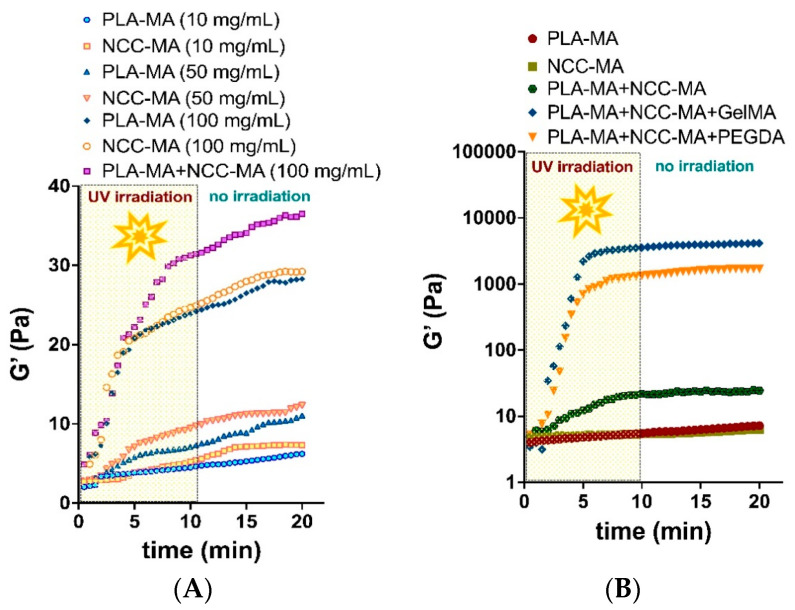
Results of rheological studies on crosslinking kinetics: (**A**) effect of particle concentration; (**B**) effect of cross-linkers (PEGDA and GelMA) on particle cross-linkers. Shown is G’ (storage modulus) evolution with time; G” (loss modulus) is not shown for the purpose of clarity. In all samples G’ > G” after exposure to UV, indicating a well-developed and solid polymer network. Samples were measured at 25 °C and were exposed to UV intensity of 1.2 J/cm^2^. The curves shown are representative ones of three measurements.

First, we studied the effect of particle concentrations on their ability to form cross-links between each other (Figure 7A and Figure 8A). Figure 7A shows the possible scheme of direct cross-linking. One can observe that the concentrations of both PLA-MA and NCC-MA played important roles in the growth of storage modulus. This is logical, because the increase in concentration affects the probability of particle interaction, which could lead to cross-linking. It was shown that for the formation of cross-links between particles, a particle concentration of at least 100 mg/mL was required. It appears that at such concentrations, particles could form a three-dimensional network; however, the mechanical properties of such networks were not enough to form stable 3D matrices. Higher concentrations of particles were not applied here, because particles could aggregate, and it was highly probable 3D printer nozzle would be clogged. it is notable that investigation of the effects of the PLA-MA and NCC-MA ratio on G’ growth showed that an 80:20 weight ratio of corresponding particles resulted in the best growth of storage modulus (Supplementary materials, Figure S4A). It should be also noted that significant after-exposure free-radical cross-linking was not observed. 

Considering the abovementioned circumstances and previous experience on the application of poly(ethylene glycol) diacrylate (PEGDA) and gelatin methacrylate (GelMA) for preparation of matrices for 3D cell culturing [51,52] we made and attempted to use such macromolecules as crosslinkers for intensification of the inter-particle interaction. The schemes of cross-linking in these cases are shown on Figure 7B,C. The effect of cross-linker concentration on G’ growth showed that concentration of 20 wt% GelMA was optimal, because increasing GelMA concentration did not result in better rheological properties (Supplementary Materials, Figure S4B).

One can observe an impressive difference in the growth of the storage modulus when using PEGDA and GelMA as crosslinking agents (Figure 8B). The values of the G’ increased by more than two orders of magnitude after 10 min of irradiation with UV lamp when crosslinking macromolecules were added to the mixture. The materials formed under such conditions seemed to be stable 3D networks. It is notable that the application of GelMA yielded a greater increase in G’ than in the case of PEGDA. This might be explained by the fact that GelMA is a multifunctional cross-linker agent, bearing several MA groups in its structure, while PEGDA is only bifunctional. Such a benefit of GelMA provides better steric accessibility of MA groups for participation in free-radical cross-linking. The greater molecular mass of GelMA as compared to that of PEGDA could be also an important factor in the observed effect.

The conducted rheological experiments led us to significant conclusions: the interparticle interaction provoked by free-radical cross-linking of methacrylate groups is generally possible at concentrations above 100 mg/mL, but does not lead to the formation of mechanically stable polymer networks. Thus, the formation of particle-based scaffolds requires the application of macromolecular cross-linker. Multifunctional cross-linkers with greater molecular mass result in better cross-linking than bifunctional ones.

### 3.4. Cryogelation and 3D-Printing: Methods for Preparation of Particle-Based Scaffolds

Rheological studies allowed us to identify the composition of the mixtures containing PLA-MA and NCC-MA particles that could be turned into reasonably stable materials. In the next stage of this study, we aimed to use such compositions for obtaining macroporous matrices via cryogelation and 3D printing.

**Figure 9:**
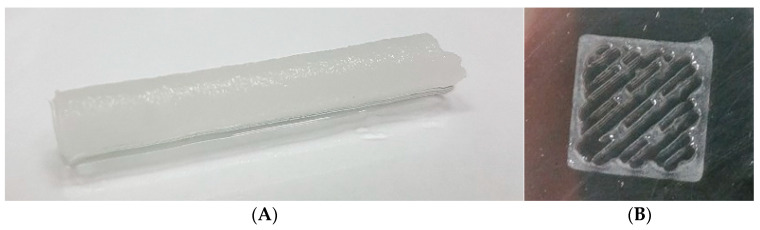
Materials obtained based on the PLA-MA/NCC-MA/Gel-MA mixture by: (**A**) cryogelation in 1 mL syringe; (**B**) 3D printing. The 80:20 weight ratio of PLA-MA and NCC-MA along with 20 wt% GelMA was applied for the preparation of these materials.

Both methods were successfully applied for the preparation of materials (Figure 9). The composition of the particle/cross-linker mixture used for cryogelation and 3D printing that was selected showed the best performance when subjected to rheological studies, and comprised PLA-MA/NCC-MA 80:20 (wt/wt) and 20 wt% cross-linker (PEGDA or GelMA).

In the case of cryogelation, the free-radical process was initiated by ammonium persulfate (APS) and 1,2-bis(dimethylamino)ethane (TEMED) RedOx initiation system. The idea of cryogelation consists in the cryo-concentration of particles and cross-linker into unfrozen liquid microphase due to the crystallization of the solvent (water), which pushes particles and cross-linker out of the crystallizing front. The action of APS and TEMED could initiate the free-radical process below 0 °C, which leads to interparticle cross-linking with the involvement of macromolecular cross-linker (PEGDA or GelMA). The described process was performed in the syringe, leading to the formation of matrices with cylindrical geometrical forms (Figure 9A).

The CAD models of the materials were designed and the DIW 3D printing of scaffolds was performed with an extrusion-type printhead and a UV lamp for photo-cross-linking of the material. A photo-initiator (Irgacure 2959, 0.1 wt%) was added to the system to provide the possibility of free-radical cross-linking reactions. Such printing parameters as printing needle diameter, pressure, and printing speed were optimized. To avoid the formation of hard gel and printhead clogging, in the case of the sample containing thermosensitive Gel-MA as an additional crosslinking agent, the optimal printing suspension temperature was also determined to be 37 °C. Uniform extrusion was observed with a printing needle diameter of 0.254 mm and length of 6.35 mm, and with pressure of 0.5 PSI or 3.45 kPa. The optimal printing speed was 5 mm/s. Three-dimensional printing and cross-linking of the sample was performed in layers (Supplementary Materials, Figure S5): after the printing of the first level of the matrix, the UV lamp was turned on, the sample was irradiated for 10 min, and then the printing process was resumed. Fully printed samples were left for one hour for the completion of free-radical reaction and drying (Figure 9B).

**Figure 10:**
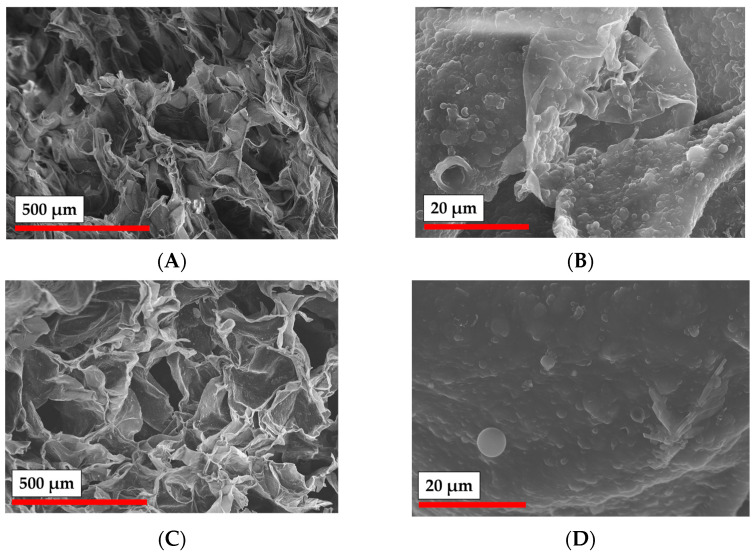
SEM images of matrices obtained by cryogelation of PLA-MA NPs with NCC-MA with different cross-linkers at different magnification: (**A**) Gel-MA (×100); (**B**) Gel-MA (×2000); (**C**) PEGDA (×100); (**D**) PEGDA (×2000).

**Figure 11:**
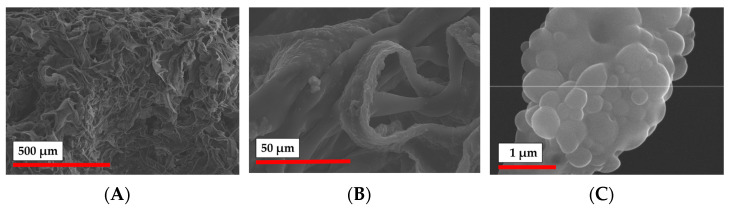
SEM images of PLA-MA NPs/NCC-MA 3D-printed matrix: (**A**) ×100; (**B**) ×1000; (**C**) ×30,000.

The morphology of samples obtained by cryogelation and 3D printing was studied by SEM (Figure 10 and Figure 11). Particle-based cryogel samples, obtained with the application of both GelMA and PEGDA, demonstrated a macroporous nature with average pore diameter of around 200 µm (according to SEM). Additionally, matrices showed good pore interconnectivity, which is important for scaffolds. Interestingly, the examination of the pore walls under a larger magnification demonstrated the fact that the particles were incorporated into the pore walls (Figure 10B,D).

Based on the results of the study of the rheology of particle crosslinking with different cross-linkers in the presence of the photoinitiator (Figure 8B), only compositions containing GelMA were used for 3D printing. The morphology of the obtained material at different magnification was studied by SEM (Figure 11). It should be noted that in contrast to the cryogelation process, the pore structure in the 3D printing was not provided by phase separation, but was governed by the designed CAD model. However, the surface of the 3D printed hydrogel scaffold was shown to be quite rough, having pores with 10 to 100 µm in diameter (Figure 11A). As in the case of cryogelated materials, the magnification of the material surface allowed the observation of the particles incorporated into the walls of the 3D printed matrices (Figure 11B,C).

Thus, both cryogelation and 3D printing approaches could be applied to form particle-based materials with the application of PLA-MA, NCC-MA, and GelMA as cross-linkers. The application of PEGDA as a cross-linker does not result in any beneficial structure. At the same time, GelMA could be considered as a more promising material, because it bears a specific arginine-glycine-aspartic acid (RGD) cell-adhesion moiety [53], which is important for the induction of novel tissue formation [11].

### 3.5. Mechanical Testing

**Figure 12:**
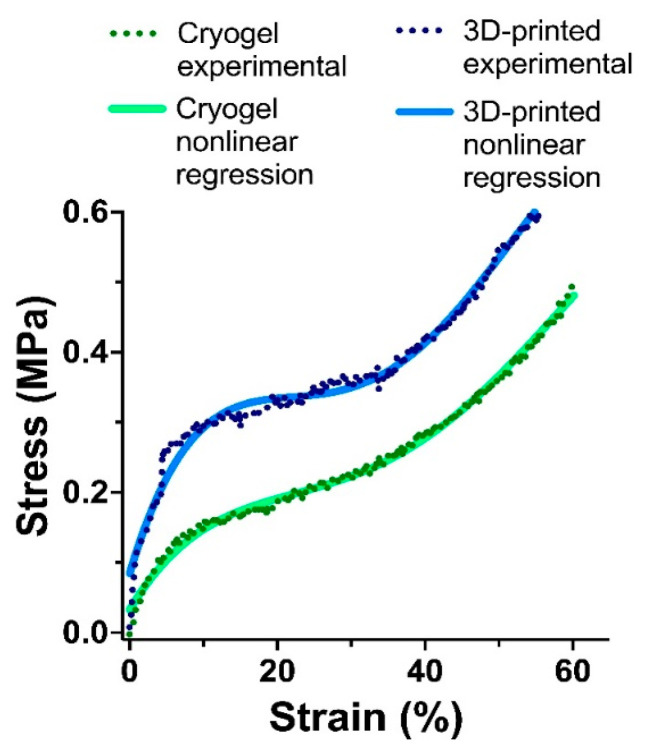
Strain–stress test curves for cryogelated and 3D-printed matrices. The compression stress was applied with initial force 0.1 N. The speed of compression was 2 mm/min. The data on the graph are representative of 3 measurements (*n* = 3).

The mechanical strength of materials is an important characteristic for scaffolds, especially in the case of the tissue engineering of hard tissues [1]. Thus, we performed a comparative study of matrices obtained by cryogelation and 3D printing based on PLA-MA/NCC-MA and GelMA as cross-linkers. The strain–stress curves obtained during sample compression are presented in Figure 12. The mechanical properties of both cryogelated and 3D printed matrices were not extraordinary and more or less corresponded to those of hydrogels. At the same time, it was obvious that the 3D printed material possessed better mechanical properties than the cryogelated one. This can be explained by the thicker walls and lower overall porosity, and hence the greater density of the printed material as compared to cryogel. It also should be mentioned here that 3D printing allowed control over the thickness of formed material walls, thus the mechanical properties could be controlled to form gradients of mechanical strength. Such material formations will be presented in our future papers. Thus, based on the above-mentioned arguments, we can propose 3D printed scaffolds as better candidates for hard-tissue engineering.

### 3.6. Cell Adhesion on Cryogelated and 3D Printed Matrices

**Figure 13:**
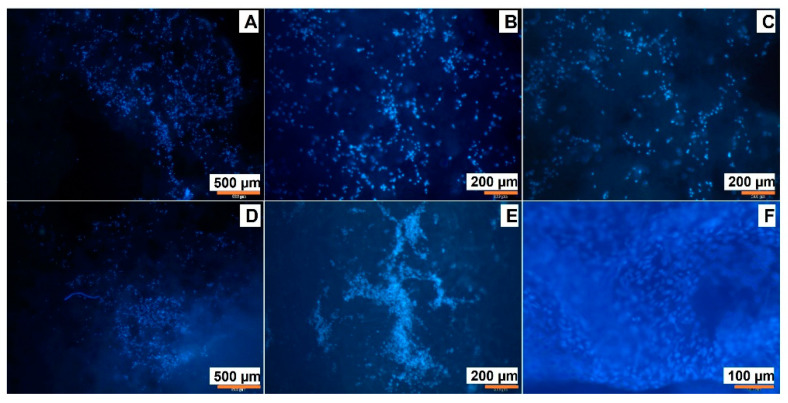
Fluorescent images of DAPI-stained mesenchymal stem cells (MSC) on the surface of: (**A**–**C**)—cryogelated matrix PLA-MA/NCC-MA + 20% PEGDA; (**D**–**F**) 3D-printed matrix PLA-MA/NCC-MA + 20% GelMA. Images were recorded at different magnification: A and D ×2; B and E ×4; C and F ×10.

Aa a final step of the current study, we performed a comparative study of MSC adhesion on the surface of the cryogelated matrices and the 3D printed ones. PLA-MA and NCC-MA ratios were chosen as based on the previous results and were 80:20 (wt/wt). In the case of cryogel, PEGDA was applied as cross-linking agent, while in the case of 3D printed material it was GelMA. To test cell adhesion matrices were incubated with MSC cells on a 24-well plate for one week in MEM. Then, the cells adhered to the matrices were visualized by DAPI-staining. One can observe (Figure 13) that the number of cells on the surface of 3D printed material seem to be greater than on the surface of cryogelated matrix.

**Figure 14:**
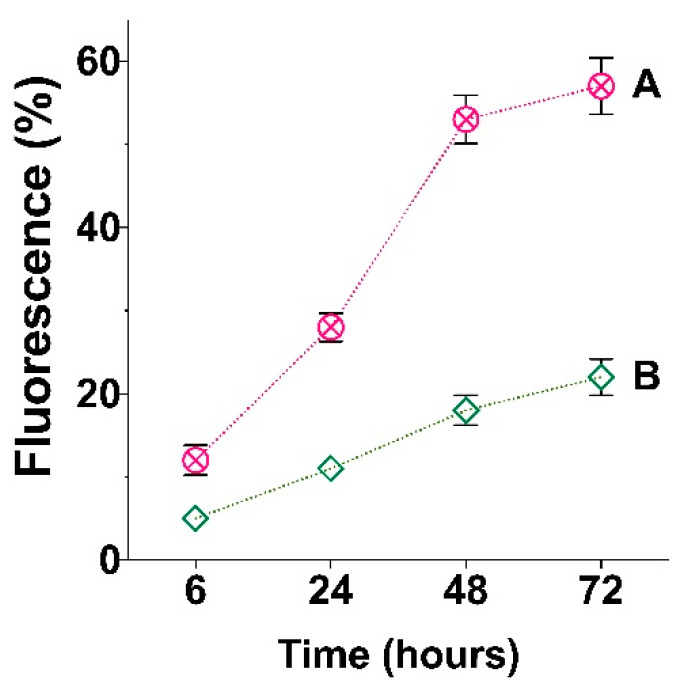
Results of fluorescence analysis of attached DAPI-labeled MSCs on the surface of: (**A**) 3D printed matrix PLA-MA/NCC-MA + 20% GelMA and (**B**) cryogelated matrix PLA-MA/NCC-MA + 20% PEGDA after 6, 24, 48, and 72 h after seeding.

In order to obtain quantitative results, we measured the intensity of the fluorescence on the surface of cryogelated and 3D printed materials with equal geometry (round discs). The obtained data (Figure 14) are in line with visual data on Figure 13 and clearly show that the number of cells attached to the 3D printed material is significantly greater than that in the case of cryogels. This observation is even more interesting given the apparently higher surface area of the cryogel matrices. It is most likely that the surface area factor was more than counterbalanced by the presence of GelMA in the printed matrices, which in turn contained specific cell adhesion moiety, namely RGD. Such a specific moiety initiates intensive cell adhesion due to signal transmission between cells [54].

## 4. Conclusions

In the presented study we were aimed to study the possibility of particle-based scaffold formation as based on PLA and NCC. In order to form covalent cross-links between these particles, we introduced methacrylate groups on their surfaces. Such modification did not greatly affect particle morphology, namely the spherical nature particles, in the case of PLA and needles in the case of NCC. Furthermore, no increasing toxicity of modified particles in relation to the MSCs and NIH/3T3 cells was observed.

The investigation of particle interaction and the ability to form three-dimensional networks under free-radical polymerization conditions showed that particles can form only very poor networks (storage modulus 30–40 Pa), when the reaction takes place only between the particles themselves. The addition of macromolecular cross-links, such as PEGDA and GelMA, in concentrations of 20 wt% significantly changed the situation, and reasonably stable networks could be formed. Such networks were characterized by a storage modulus as large as 2000–4000 Pa. The developed particles/cross-linker compositions were successfully applied for the formation of materials via cryogelation/cryopolymerization technique and DIW 3D printing with photo-curing. The study of the material morphology showed that both types of materials, namely cryogels and 3D printed matrices, contained particles in their structure. Mechanical tests showed that 3D printed materials possessed greater strength than cryogelated ones. The study of MSCs adhesion clearly showed that application of GelMA as a cross-linking agent is highly preferable if one wants to initiate specific cell adhesion. The fluorescence of DAPI-stained cells was three times higher when GelMA was used as the particle cross-linker.

The results of this study could be useful for the preparation of 3D scaffolds with the application of particles. Further attempts should be made to generate gradient scaffolds via printing with different printheads and extruders loaded with different particles. Thus, we can say that particles are another prospective component in the toolbox for creating scaffolds that will effectively support the formation of new tissue both in vitro and in vivo.

## Figures and Tables

**Table 1:** Effect of particle modification on particle diameter and surface charge.

Sample #	D_H_ (DLS), nm	ζ-Potential, mV	D (TEM), nm	D (NTA), nm
PLA MPs	258 ± 74	−45 ± 7	210 ± 70	263 ± 85
PLA-MA MPs	347 ± 129	−33 ± 8	290 ± 120	443 ± 181
NCC	95 ± 18	−39 ± 11	not determined	121 ± 30
NCC-MA	110 ± 24	−36 ± 5	not determined	182 ± 51

## Data Availability

Data available within the article or its Supplementary Materials.

## Supplementary Materials

The following supporting information can be downloaded at: https://www.mdpi.com/article/10.3390/polym15030651/s1, Figure S1. Raman spectrum of PLA (green line) and PLA-MA (blue line). Raman spectra were recorded with T64000 Horiba Scientific spectrometer (Kyoto, Japan). Figure S2. Non-methacrylated and methacrylated particles characteristics: (A)—PLA and PLA-MA; (B)—NCC and NCC-MA. Figure S3. The scheme of rheological experiment, which was applied for the testing of interparticle cross-linking. Figure S4: Results of rheological experiment: effect of PLA-MA and NCC-MA weight ratios (A) and Gel-MA concentration (B) on storage modulus increase during 10 min of exposure to UV-light and thereafter. Figure S5: The scheme of 3D printing with the application of particles as photo-curable ink.
