# Supplementary material for: Poly(lactic acid) and Nanocrystalline Cellulose Methacrylated Particles for Preparation of Cryogelated and 3D-Printed Scaffolds for Tissue Engineering
Source: Polymers (Basel). 2023 Jan 27;15(3):651. doi: 10.3390/polym15030651 (PMC9920993; doi:10.3390/polym15030651)
Supplement: Supplementary file 1 [file polymers-15-00651-s001.zip › polymers-2121055-supplementary.pdf]

Supplementary Materials

Poly(lactic acid) and nanocrystalline cellulose methacrylated particles for preparation of 3D printed hydrogel scaffolds for tissue engineering

Mariia Leonovich [1], Viktor Korzhikov-Vlakh [1], Antonina Lavrentieva [2], Iliyana Pepelanova[2], Evgenia Korzhikova-Vlakh [1,3], Tatiana Tennikova [1*]

[1] Institute of Chemistry, Saint Petersburg State University, Peterhoff, Universitetskii pr. 26, 198504 St. Petersburg, Russia; v.korzhikov-vlakh@spbu.ru
[2] Institute of Technical Chemistry, Gottfried-Wilhelm-Leibniz University of Hannover, 30167 Hannover, Germany; lavrentieva@iftc.uni-hannover.de
[3] Institute of Macromolecular Compounds, Russian Academy of Sciences, St. Petersburg 199004, Russia; vlakh@mail.ru

* Correspondence: tennikova@mail.ru

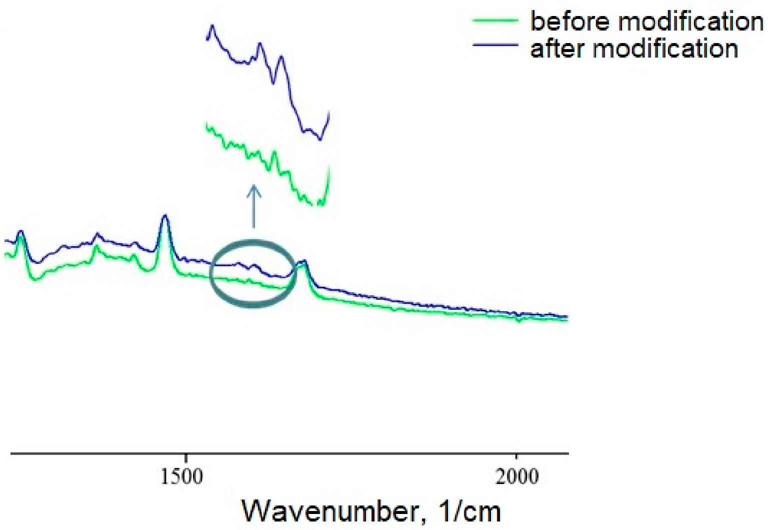

Figure S1. Raman spectrum of PLA (green line) and PLA-MA (blue line). Raman spectra were recorded with T64000 Horiba Scientific spectrometer (Kyoto, Japan).

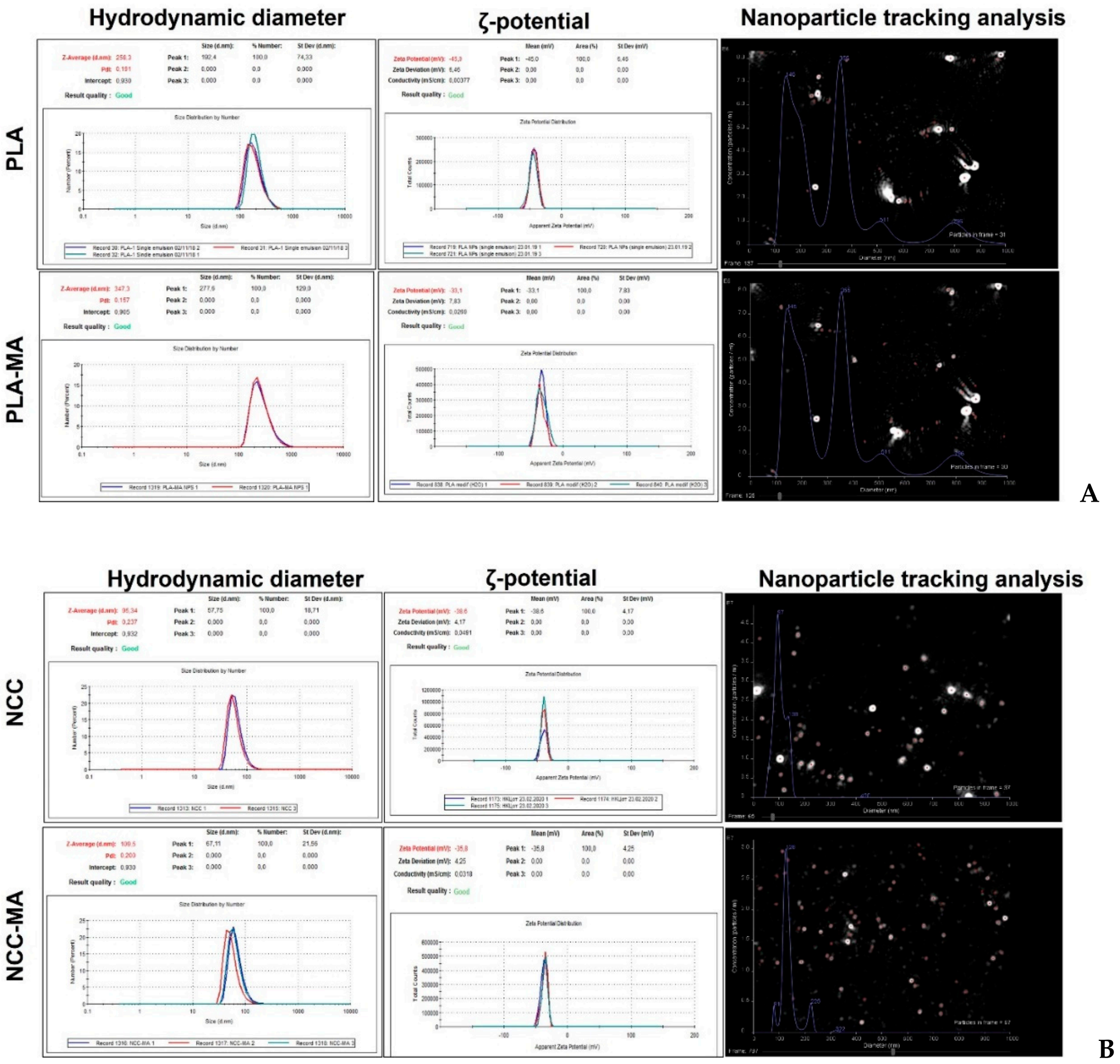

Figure S2. Non-methacrylated and methacrylated particles characteristics: (A) – PLA and PLA-MA; (B) – NCC and NCC-MA.

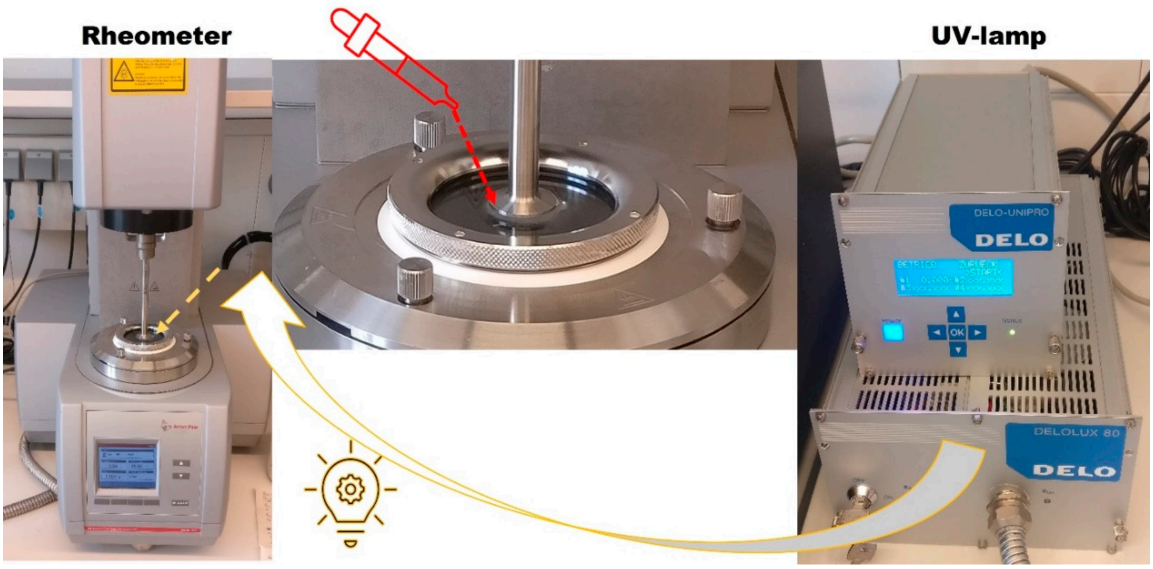

Figure S3. The scheme of rheological experiment, which was applied for testing of interparticle cross-linking.

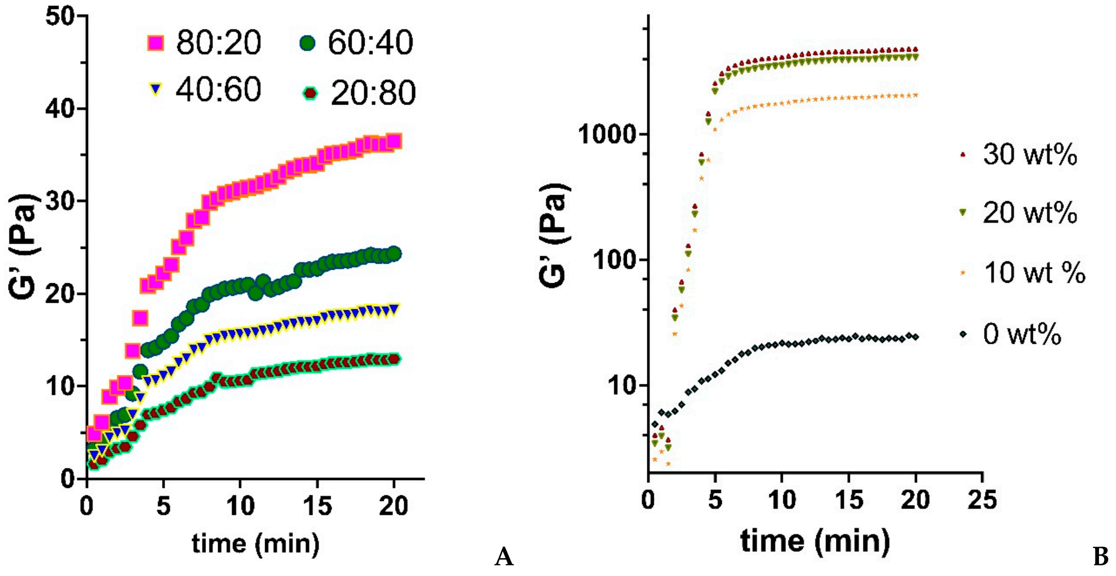

Figure S4. Results of rheological experiment: effect of PLA-MA and NCC-MA weight ratios (A) and Gel-MA concentration (B) on storage modulus increase during 10 min of exposure to UV-light and thereafter.

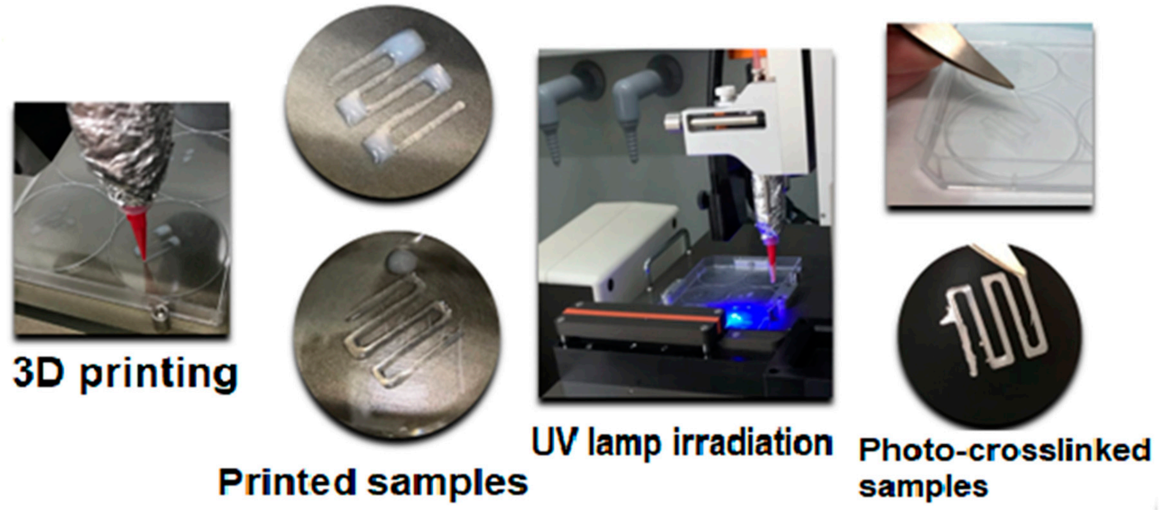

Figure S5. The scheme of 3D printing with application of particles as photo-curable ink.
